# IMU-Based Bilateral Kinematic Waveform Analysis Associated with Shoulder Pain History in Competitive Swimmers

**DOI:** 10.3390/s26206565

**Published:** 2026-10-16

**Authors:** Ben Gronbach, Kyle Capili, Seyedeh Fatemeh Khatami Firoozabadi

**Affiliations:** Bioengineering Department, University of the Pacific, 3601 Pacific Ave, Stockton, CA 95211, USA; b_gronbach@u.pacific.edu (B.G.); k_capili@u.pacific.edu (K.C.)

**Keywords:** injury, bilateral, motion analysis, swimming

## Abstract

Shoulder injuries are one of the most common overuse problems in competitive swimming, yet objective, field-deployable methods for quantifying pain-related movement alterations remain limited. This study investigates whether bilateral IMU sensors placed on the upper humerus can identify kinematic bilateral waveform similarities tied to a history of shoulder pain in swimmers. In this study, thirty-four NCAA Division 1 collegiate swimmers and water polo players completed a standardized swimming test and were classified into moderate/severe pain (*n* = 22) and none/mild pain (*n* = 12) groups based on self-reported shoulder pain scores. Kinematic features were extracted and evaluated using statistical tests and machine learning classification. The none/mild pain group showed a higher peak left-right shoulder cross-correlation across all axes (0.85 vs. 0.78, *p* = 0.002). The best-performing model Classification accuracy for distinguishing between moderate/severe pain and none/mild pain conditions achieved a balanced accuracy of 70.6% (24/34 participants), with a balanced accuracy of 69.7% (95% CI [52.7–85.2%]) and an ROC AUC of 0.731 (95% CI [0.557–0.890]), suggesting that personalized machine learning approaches may be necessary to improve individual-level classification performance. Further investigation of intra-subject associations between shoulder pain history and kinematic bilateral waveform similarities is warranted to better understand their relationship and determine potential causal mechanisms.

## 1. Introduction

Despite the classification of swimming as a non-contact sport, debilitating injuries are undeniably frequent, with a swimmer’s shoulder being the most injured joint. Among competitive swimmers, reported rates of injured shoulders vary from 40% to 91% across different studies [1,2,3,4]. Considering the sheer yardage that swimmers complete in their training schedules, this high frequency of injuries does not come as a surprise. A study conducted found that at NCAA Division 1 programs, swimmers perform approximately 6000 to 10,000 yards per day [5], with typical programs of training 6 days per week [6]. This rigorous training schedule puts an immense amount of biomechanical stress on the shoulder, which contributes to about 90% of forward propulsion in the front crawl stroke [1]. The repetition and frequency of the overhead motion in a swim stroke degrades the shoulder over time, often leading to rotator cuff impingement, tendinitis, or tearing of the labrum. It is known that shoulder pain is correlated with the number of years spent in competitive swimming, and swimmers completing higher weekly volumes of swimming are more likely to experience shoulder pain [7,8]. Shoulder injuries are often exacerbated by issues of muscular imbalances, improper stroke technique, and excessive joint laxity [1,9,10]. Identifying these predictors prior to injury can help preserve shoulder health, since early recognition and treatment of overuse injuries support better outcomes and more effective prevention [2,11]. Raffini et al. showed that participating in a physiotherapy session prior to a swimming workout reduced the effects of fatigue, maintaining a more consistent biomechanical load distribution [12]. Early discovery of shoulder problems allows coaches to tailor the workload to the individual needs of their athletes, which can reduce injury risk and allow swimmers to continue competing at a high level.

Most commonly, coaches will observe their swimmers and adjust their technique based on their own personal experience and knowledge [13]. This method is easy, low cost, and requires no setup. However, coaches’ judgements are subjective and unstandardized and carry no quantitative data behind their observations. Traditional quantification methods are video-based, which requires setting up a system of cameras above or below the water to acquire features such as speed, body position, and joint angles, among others [14]. However, these systems are expensive and require a high and careful degree of calibration and installation. Turbulence in the camera’s view by external factors such as refraction, the water-air interface, and splashing make data processing difficult [15]. Additionally, camera systems require a participant to stay within a certain field of view, potentially constraining the movement of a study participant [16].

Wearable sensor-based motion capture systems represent a cheap and portable alternative. Inertial Measurement Units (IMUs) have emerged as a strong competitor. IMU setup is very minimal compared to optical systems and is not limited by any spatial boundary when collecting data. It has been found that measuring joint angles in swimming with IMUs displayed a comparable accuracy to an optical-based system [14].

IMUs have been increasingly applied in swimming biomechanics to provide objective, continuous measurement of technique parameters that are difficult to observe visually, particularly for movements occurring beneath the water surface. They have been used to quantify swim velocity, stroke count, distance per stroke, body roll, and body balance [17], as well as quantify left-right stroke asymmetry [18], among swimmers during in-water testing.

IMUs have also been used in clinical rehabilitation settings to quantify gait parameters by measuring gait asymmetry, cadence, stride length, and stance and swing phase durations [19]. Furthermore, IMUs have been applied to the clinical assessment of shoulder pathology [20].

The use of IMUs to quantify aspects of shoulder pain in swimmers has increased in recent years. One research group found measurable differences in the amplitude of internal/external rotation in swimmers with injured shoulders after fatiguing exercises and suggested that it may be due to muscle compensation [12]. This increase in amplitude was reduced following physiotherapy treatment, suggesting that IMU-derived kinematic measures can detect meaningful changes in shoulder mechanics across the course of rehabilitation and shoulder range of motion [21]. However, they limited their use of IMUs to measuring only out-of-the-pool exercises, therefore not investigating the kinematics of the swim stroke movement itself. Another study used sacrum- and hip-mounted IMUs and found that swimmers with no shoulder pain displayed a higher hip-level roll than swimmers experiencing shoulder pain, suggesting that pain-related differences can be quantified through kinematics [3].

IMUs are used in different studies for movement analysis [17,18,19,20]; however, there is limited literature using IMUs to profile biomechanical abilities during swimming [22], and no prior literature has demonstrated IMU-derived biomechanical features capable of differentiating history of shoulder and upper arm pain status using deltoid-mounted IMUs. In this study, we investigate whether deltoid-mounted IMU features capture pain-related movement dissimilarities that discriminate between swimmers with and without shoulder pain. Since shoulder pain frequently stems from an imbalanced stroke technique and load distribution, we hypothesized that swimmers with shoulder pain will demonstrate noticeably lower bilateral waveform similarities in their upper-arm stroke kinematics.

## 2. Materials and Methods

### 2.1. Study Design

A cross-sectional observational study is presented with the purpose of investigating kinematic signals on swimmers with shoulder injuries. The study was done at the University of the Pacific under approved IRB (#IRB2026-47). The data were collected at the Douglass M. Eberhardt Aquatic Center at the University of the Pacific in Stockton, California. All participants were asked to fill out a questionnaire before the study (Supplementary Materials). The overall workflow is outlined in Figure 1 and will be further detailed throughout this section.

### 2.2. Participants

Study participants were recruited from the University of the Pacific’s varsity swim and water polo teams, comprising seven women’s swimmers, ten women’s water polo players, seven men’s swimmers, and ten men’s water polo players. Data was collected from 40 subjects. Six datasets were excluded from analysis due to data recording errors within the sensors, yielding a final analytic sample of 34 participants (17 females, 17 males).

Male participants reported a mean height of 184.3 ± 10.0 cm and body mass of 86.7 ± 8.2 kg; female participants reported a mean height of 165.6 ± 6.3 cm and body mass of 64.1 ± 8.5 kg. All participants reported more than five years of competitive swimming experience.

Immediately prior to testing, participants completed a shoulder pain questionnaire reporting injury history, pain location, pain severity, and history of labrum tears. Most participants (20/34, 59%) reported a prior history of shoulder injury. Of these, seven (20.6% of the total sample) reported a confirmed labrum tear. Figure 2 summarizes the demographic and anthropometric characteristics of the participants, stratified by sex (female and male). The figure presents the reported side of breathing and the presence of labral tear, as well as mean height and weight and estimated upper-arm, forearm, and hand measurements. For the continuous measures, error bars represent one standard deviation (SD) from the mean. Segment masses were estimated using the sex-specific regression equations of de Leva [23], based on the modified Zatsiorsky–Seluyanov model.

*Y* = *B*_0_ + (*B*_1_ × *m*(*Weight in* kg)) + (*B*_2_ × *h*(*Height in* cm)) (1)

where *Y* is the target inertia parameter being calculated for a specific body segment. Segment Mass, (*m_i_* in kg), m was participants’ total Body Mass (in kg), and *h* was participant’s total Height (in cm). *B*_0_ was a constant intercept offset for the segment, *B*_1_ was a weighting coefficient for total Body Mass (*m*), and *B*_2_ was a weighting coefficient for total Height (*h*). Using sex-specific segment inertial parameters [23], the higher upper-limb segment masses observed in male swimmers (Upper Arm ~ 2.35 kg, Forearm ~ 1.40 kg) impose substantially greater passive rotational inertia on the glenohumeral joint during the recovery phase of the freestyle stroke. This elevated moment of inertia increases resistive torques, requiring higher stabilizing force from the supraspinatus and infraspinatus, which may contribute to rotator cuff fatigue and subacromial impingement. This may account for the higher prevalence of moderate/severe shoulder pain reported among female participants compared to males (15 vs. 7, respectively).

Pain severity was assessed using a four-point ordinal scale in reference to the peak of their competitive training season: 0 (no pain), 1 (pain present but does not affect stroke mechanics), 2 (pain that may affect stroke mechanics), and 3 (pain that definitively affects stroke mechanics). The full sample reported a mean rating of 1.74 ± 0.86; only four participants reported no shoulder pain (rating of 0). To account for the high level of individual subjectivity from participants reporting pain, the 4-bin pain categories were merged into two categories to facilitate further analysis. Participants reporting a 0 or 1 were classified as 0 (none/mild pain), and participants reporting either a 2 or 3 were labeled as 1 (moderate/sever pain). Using the new categories, 12 participants (35%) were classified as having none/mild pain, and 22 (65%) were classified as having moderate/severe pain. Symptoms of pain on the day of testing were not recorded. Measuring shoulder pain history rather than pain on the day of the examination allows for more accurate insights into the nature of participants’ shoulders. A study conducted by MacFarlane et al. found that only 67% of patients with chronic shoulder pain were experiencing pain on the day of examination [24].

Participants with a history of shoulder injury reported significantly higher (*t*-test, *p* < 0.01) mean pain ratings (2.05 ± 0.76) compared to those without (1.29 ± 0.83). Regarding pain laterality, 13 participants reported right-sided pain, 5 reported left-sided pain, and 4 reported bilateral pain. Twenty-two participants reported breathing to the right and 12 to the left.

In 22 participants labeled as the moderate/severe pain group, 17 (77%) reported experiencing pain on their preferred breathing side, whereas 5 (23%) did not. Pain-group membership did not differ significantly by sport (swimmers 8/14, 57%; water polo 14/20, 70.0%), indicating that the moderate/severe pain and none/mild pain groups were not confounded by primary sport.

### 2.3. Sensor and Hardware Placement

Two MetaMotionRL [25] inertial measurement units (IMU) manufactured by MbientLab Inc. (designed in CA and assembled/manufactured in USA, China and Republic of Korea) were attached to each participant during testing with a sampling rate of 200 Hz. Sensors were secured to the lateral deltoid tuberosity using kinesiology tape in a manner that did not restrict shoulder range of motion.

Prior to use, each sensor was housed in a custom waterproof enclosure consisting of a 3D-printed case designed to conform to the sensor contours (Figure 3), fabricated with six shell layers of PLA filament at 20% infill density. The exterior of each case was treated with Gorilla Clear Waterproof Spray and Sealer, and the seal was sealed with a layer of PTFE thread seal tape followed by a layer of duct tape. The case was tested to ensure that it is waterproofed, and the case was sealed appropriately in the benchmark test.

In the next step, the sensors were placed on both the left and right shoulders of participants. Axis configurations for both the gyroscope and accelerometer components are shown in Figure 4 (Figure 4a which shows placement of the sensor, is generated by ChatGPT-5). Sensors were placed in an orientation such that all positive and negative directions for the X, Y, and Z axes were aligned. However, since bilateral limbs move laterally in a mirrored image, the same anatomical movement will be represented by flipped axes. A lateral motion on the left arm follows the negative Z axis, whereas a lateral movement on the right arm moves along the positive Z axis.

### 2.4. Experimental Protocol

After filling out the questionnaire, participants were instructed to perform catch-up freestyle swimming for eight pool lengths in a 25-yard pool (200 yards total) at approximately 70% perceived exertion. Swimmers were instructed to use bilateral breathing patterns, minimize wall push-offs, and rest for 5 s between lengths. The block diagram of the experiment set up is shown in Figure 5 (Block diagram was generated by ChatGPT-5 based on the experiment setup).

## 3. Data Processing and Feature Extraction

After collecting data from participants, the workflow was implemented using core Python packages (Python version 3.1.3), specifically NumPy, pandas, and scikit-learn. To process the raw kinematic data into comparable features, inter-sensor lags and sensor noise needed to be removed. Next, the data needed to be trimmed and segmented into laps to isolate the swimming subset from idle periods. Therefore, each individual cyclic motion stroke needs to be detected to extract individual stroke features. Below is the breakdown for each subprocess. Noise identification and stroke detection were partially performed manually. All manually annotated parameters were documented and are available to download (Supplementary Materials).

### 3.1. Signal Preprocessing

Raw IMU signals were processed to extract time- and frequency-domain biomechanical features. Swimming bout start and end time boundaries were identified manually through visual inspection of the raw signals. All manual threshold tuning was done blind with respect to subject pain status to prevent data contamination. These manual tuning parameters have been saved and are available to ensure full reproducibility of the procedure.

To correct for inter-sensor timing misalignment, a synchronization jerk test was performed prior to each session, during which both sensors were simultaneously subjected to a sharp, identical motion to generate coincident high-amplitude spikes, serving as a reference point between sensors (Figure 6). Cross-correlation analysis of the jerk-test region in the X axis was then used to estimate the temporal offset between sensors, and the right-sensor signal was shifted accordingly to align both signals to a common temporal reference frame prior to further analysis. Signals were filtered using a 4th-order low-pass Butterworth filter, with a cutoff frequency of 3 Hz to remove excess sensor noise, allowing for accurate lap and stroke segmentation.

### 3.2. Stroke Segmentation Pipeline

Lap detection was performed using the Gyroscope Z-axis signal, as this axis most reliably distinguished swimming from resting periods. A non-overlapping sliding window approach was applied, with window lengths individually optimized per subject. A window was classified as swimming if the peak rotational velocity exceeded a subject-specific threshold; these thresholds were manually determined through visual inspection and saved to ensure replicability. Consecutive swimming windows were merged into continuous lap segments, and any resulting segment shorter than 4 s was discarded. Detection was performed independently on both the left and right shoulder sensors using manually inputted parameters.

Stroke timings were identified within each lap using a smoothed and demeaned Gyroscope Z-axis signal (Figure 7). Smoothing was achieved via a 201-sample centered moving average, after which falling zero-crossings were detected and marked as individual stroke timings.

Timings were computed on both left and right shoulder Gyroscope Z-axis data and transferred to all other axes.

### 3.3. Feature Extraction

During the exploratory phase, a comprehensive set of time- and frequency-domain features was extracted from all three axes of the accelerometer and gyroscope signals. Statistical analyses were subsequently performed to evaluate the relevance of the extracted features. Based on these analyses, only the relative shoulder correlation (PeakCorr) was retained for subsequent analysis (Table 1).

Relative shoulder correlation (PeakCorr) was calculated on a per-lap basis and then subsequently averaged across the entire test session. The left and right signals are truncated to the shorter segment length. The signals were subsequently standardized using z-score normalization, and cross-correlation was computed across all valid time lags to quantify their temporal correspondence. The peak lag was defined as the time lag at which the absolute cross-correlation reached its maximum value. Cross correlation was calculated using:

(2) $$R_{\mathrm{xy}}\left[k\right]=\frac{1}{{(N.\sigma }_{x\;}{.\sigma }_{y\;})}\;\sum_{n=\max(0,-k)}^{\min(N-1,N-1-k)}\bar{x}\left[n\right].\;\bar{y}\left[n+k\right]\;$$

where N was each lap length and k was the discrete time-shift parameter between [−(N − 1), N − 1]. *N*.*σ_x_*.*σ_y_* was normalization factor-based variance of data from each IMU sensor to ensure that *R_xy_*[*k*] ∈ [−1, 1]. $\bar{x}\left[n\right]$ and $\bar{y}\left[n\right]$ are the zero-mean amplitude values for collected data from left and right IMU sensors.

### 3.4. Statistical Analysis

The relationships of IMU-derived features and subject-level pain status were investigated to assess feature importance. Due to the class imbalance and small group sizes of the cohort, the significance of features was investigated using the Brunner-Munzel test statistic (BM), which does not assume normal distribution or equal variance between groups [22,23]. The significance level was determined at 5% (*p* < 0.05). Effect size was quantified using the stochastic superiority estimate $\hat{p}=P$ (moderate/severe pain < none/mild pain), where $\hat{p}>0.5$ indicates the none/mild group tends toward higher values [22]. The three features aggregate PeakCorr features in question (Gyr_PeakCorr, Acc_PeakCorr, and Ovr_PeakCorr) were tested via the BM test. A Holm-Bonferroni correction was applied to the *p*-values to account for the possibility of false significance detections during multi-variable significance testing. The reported *p*-values are corrected accordingly [26].

### 3.5. Classification Modeling

Various machine learning models were trained and tested on these data to investigate whether pain classes were distinguishable via classification modelling. Since this study only incorporates 34 subjects with an uneven class balance (22 pain, 12 no pain), these models serve solely as exploratory vessels and are in no way portrayed as deployable stage algorithms.

All models were trained on leave-one-out cross-validation (LOOCV) to account for the small subject population. Elastic Net-regularized logistic regression (Elastic Net) and Support Vector Machine (SVM) classifiers were each trained and evaluated on the three aggregate PeakCorr features (Gyr_PeakCorr, Acc_PeakCorr, and Ovr_PeakCorr). Model performance was assessed using accuracy, balanced accuracy, and area under the receiver operating characteristic curve (ROC AUC). Accuracy was defined as the proportion of correctly classified participants out of the total sample. Because the dataset was imbalanced between moderate/severe pain and none/mild pain groups, balanced accuracy (the average recall across both classes) was used as the primary metric to account for unequal class sizes. ROC AUC was calculated to evaluate the model’s ability to discriminate between the two classes across all classification thresholds Balanced accuracy and ROC AUC, along with their 95% confidence intervals (CIs), were estimated using stratified bootstrap resampling (2000 resamples) of the pooled out-of-fold predictions. Participants with moderate/severe pain and those with none/mild pain were resampled separately with replacement to preserve class proportions. The 95% CIs were defined by the 2.5th and 97.5th percentiles of the resulting bootstrap distributions. Hyperparameters, such as L1 Ratio, number of estimators, or c values, were tuned via 5-fold grid search cross-validation nested within each LOOCV fold, optimizing for balanced accuracy between the two classes.

## 4. Results

### 4.1. Biomechanical Feature Analysis

The peak Left-Right shoulder correlation features (PeakCorr) were shown to be highly discriminating features between moderate/severe pain and none/mild pain groups (Figure 8). All aggregate features were significant with respect to pain, according to a Brunner-Munzel test with corrections. (see Table 2). The aggregate correlation value calculated by the mean of gyroscope and accelerometer X, Y, and Z axis peak correlations (Ovr_PeakCorr) was the strongest discriminator (BM = −3.80, *p* < 0.001). Splitting ties equally, the probability that a random moderate/severe pain-group subject had a lower Ovr_PeakCorr than a random none/mild pain-group subject was $\hat{p}$ = 0.79, 95% CI [0.63, 0.95]. In all cases, the none/mild pain group had a higher median peak correlation compared to the moderate/severe pain group, suggesting the none/mild pain group displayed a higher level of bilateral shoulder waveform similarity compared to the moderate/severe pain group.

### 4.2. Model Classification Accuracy

The different models were evaluated with three different metrics (accuracy, balanced accuracy, and ROC AUC) on their ability to classify participants as either moderate/severe vs. none/mild pain (Table 3). Overall, the elastic net model displayed the highest accuracy among both groups in all three metrics.

It is important to note that due to the class imbalance (22 moderate/severe pain participants vs. 12 none/mild pain participants), a model guessing all participants as the majority class would score an accuracy of 64.7%, a balanced accuracy of 50%, and an ROC AUC of 0.5. The SVM model slightly beats out an all-pain guessing baseline in balanced accuracy and ROC AUC but obtained worse results in overall accuracy. The elastic net model trained on PeakCorr features is the only model that outperforms this baseline on all three metrics.

Figure 9 shows a confusion matrix evaluating an Elastic Net model trained to classify participants pain history into two defined categories. Out of 12 actual “None/Mild Pain” instances, the model correctly identified 8 (66.7%) while misclassifying 4 (33.3%) as moderate or severe. For the 22 actual “Moderate/Severe Pain” cases, it correctly identified 16 (72.7%) and misclassified 6 (27.3%) as mild. Across all 34 total test samples, the model achieved an overall accuracy of approximately 70.6%, demonstrating slightly better sensitivity for detecting moderate to severe pain (72.7%) than specificity for none or mild pain (66.7%).

## 5. Discussion

The peak left-right correlation values across all axes represent some of the most discriminating variables between moderate/severe pain and none/mild pain groups. A higher cross-correlation score means that the left and right signals are more similar in shape to each other. In our data, a higher PeakCorr value is representative of a higher left-right shoulder kinematic waveform similarity in the swimmer’s stroke.

Across all axes, the none/mild pain group displayed a higher correlation value compared to the moderate/severe group. These data indicate that a higher degree of left-right shoulder waveform dissimilarity is more prominent in study participants experiencing moderate/severe. This may be due to the biomechanical load distribution of an uneven stroke technique. An asymmetric stroke may lead to a higher degree of joint stress placed on one shoulder compared to the other. This imbalance would lead to a gradual degradation of the shoulder joint, which may cause the swimmer to experience pain in their stroke. Conversely, the opposite conclusion could be reached given these newfound data. It is possible that a swimmer experiencing a high degree of shoulder pain may subconsciously or intentionally alter their stroke with the purpose of redirecting mechanical stress away from the injured joint. Both explanations are possible given this result. To determine the true causation of this relationship, a longitudinal study measuring peak left-right correlation over time should be conducted. A comparison of swimmers’ peak left-right correlation before and after an injury would provide clearer insight into this question.

A study by Vila Dieguez et al. (2022) [3] utilized a similar methodology to the present study by splitting participants into pain and no-pain groups to identify kinematic differences with chest- and hip-mounted accelerometers. They identified no significant differences in shoulder body roll between swimmers with and without shoulder pain but reported significant differences in hip body roll, suggesting that pain may be associated with alterations in swimming biomechanics. In contrast, this study identified shoulder rotational kinematic waveform dissimilarity, quantified as left-right shoulder peak correlation, as one of the strongest discriminative features between moderate/severe and none/mild pain groups. Collectively, these findings may indicate that pain-related biomechanical alterations are more readily detectable through localized shoulder kinematics than through global measures of shoulder body roll alone.

The accuracy of the classification models should be interpreted entirely as preliminary. With 34 participants and no external validation set, the estimates carry wide confidence intervals. The highest-performing model was the Elastic Net when trained on PeakCorr features, which edged out the majority-guessing baseline in all three metrics. This suggests that while there may be some discriminating signal in this data, there is not a clear separation between moderate/severe pain and none/mild pain groups. While PeakCorr has been shown to be a strong indicator of pain at the group level, it does not straightforwardly yield strong individual-level classification. Whether PeakCorr-based classification can be developed into a clinically useful screening tool remains an open question requiring larger, prospectively validated cohorts.

A longitudinal study is needed to determine whether within-subject changes in PeakCorr track a swimmer’s recovery from shoulder problems and whether the metric is sensitive enough to detect meaningful change at the individual level. Such a design would also resolve the causal direction that the present cross-sectional data cannot: whether reduced bilateral waveform similarity precedes the onset of pain or arises as a compensatory response to it. This distinction has direct practical consequences. If bilateral waveform dissimilarity is a precursor, technique interventions aimed at improving waveform similarity may hold preventive value; swimming coaches could optimize swimmers’ techniques to improve correlation, thus potentially lowering the chance of injury. However, if it is a consequence of pain, encouraging waveform similarity could increase loading on an already symptomatic joint. Until that question is resolved, PeakCorr is best positioned as an objective quantity to supplement, but not to replace, the subjective observation currently used to monitor swim technique.

A limitation of this study is that the target variable used was a self-reported pain score rather than a clinical diagnosis of the shoulder. Pain is a subjective and uniquely personal experience, and therefore there was likely some degree of inter-subject variance in the collected pain scores, whereas a clinical diagnosis provides an objective divide between participants. Furthermore, shoulder pain was assessed as a history-based phenotype rather than as current symptom status on the day of testing. This was intentional. Day-to-day shoulder pain fluctuates with recent training load, recovery, and other short-term factors, so a single same-day rating would introduce substantial variability unrelated to a participant’s underlying shoulder condition. A history-based classification is more stable and more consistent across participants, and it targets the persistent movement adaptations hypothesized to accompany recurrent shoulder pain. Therefore, the scope of this study is limited, as it addresses kinematic patterns associated with shoulder-pain history rather than acute symptoms at the time of the recording. Moreover, the shoulder is an anatomically complicated joint, and therefore pain can manifest itself in a multitude of ways. This study defines shoulder pain as a general term rather than classifying different pathologies. Pain can potentially manifest itself in a swimmer’s kinematic stroke in different ways. For example, some participants experiencing pain may reduce rotation in one shoulder to protect the joint, whereas another participant may experience pain because of an overrotation in one shoulder compared to the other. In this case, both participants experience pain, yet their kinematics would vary greatly from one another. Another limitation of this study is the cross-sectional nature, which limits the ability to investigate intra-participant variation. Additionally, this study had a large class imbalance between moderate/severe pain (*n* = 22) and none/mild pain (*n* = 12), which reduces statistical power to detect group differences. Furthermore, the relatively small total participant size (*n* = 34) could be increased to increase generalizability to the population. A limitation of the study protocol was that subjects were instructed to swim at a 70% effort level rather than a controlled interval pace, which may have led to a variation of speeds across different subjects. This may have caused changes in feature values, specifically acceleration and velocity peaks, and frequency-derived features. The instruction to breathe bilaterally and to swim a catch-up style of freestyle may have unintentionally altered participants’ strokes by encouraging them to swim in a manner that differs from their typical stroke technique.

As previously stated, further investigations should include a longitudinal study to set up an individualized baseline, enabling temporal causal inference rather than cross-sectional association. Additionally, using a larger cohort of participants could expand the scope of this study, as the rotational patterns of the shoulder are complex and may not be fully represented by the current sample. Furthermore, multi-sensor integration combining kinematic data from IMUs with muscle electrical activity from sEMGs could provide better insight into the way pain is manifested within a swimmer’s stroke technique by allowing for investigation into potential pain-related stroke-altering compensation.

## 6. Conclusions

In this study we identify bilateral shoulder correlation, measured with upper-arm mounted IMUs, as a discriminator for shoulder pain status in swimmers. This study was conducted to explore the hypothesis that swimmers experiencing shoulder pain would display noticeably different rotational kinematic bilateral waveform similarities compared to a comparison group. Consistent with this hypothesis, it was found that swimmers experiencing shoulder pain displayed a significantly lower level of peak left-right shoulder correlation. These group-level differences warrant future evaluation of wearable shoulder-mounted IMU-derived kinematics as a framework for identifying injury-related movement patterns and monitoring rehabilitation progression in swimmers. However, it remains unknown whether reduced bilateral waveform similarity precedes shoulder pain or arises as a compensatory response to it. Further investigation is warranted to better characterize this relationship. It is recommended that the following studies be longitudinal rather than cross-sectional to establish intra-subject comparisons between kinematics and pain levels.

## Figures and Tables

**Figure 1:**
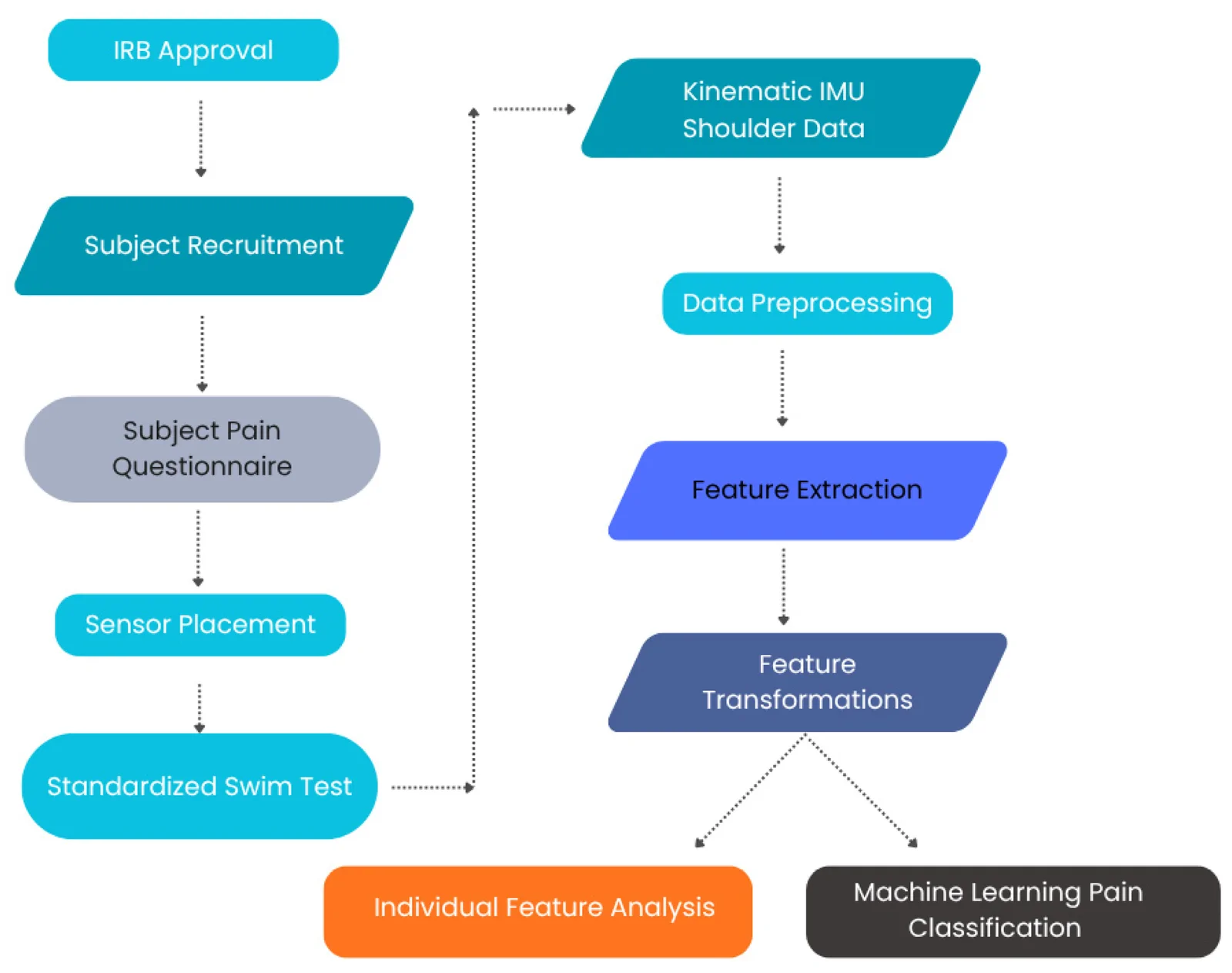
Workflow Diagram of the Study.

**Figure 2:**
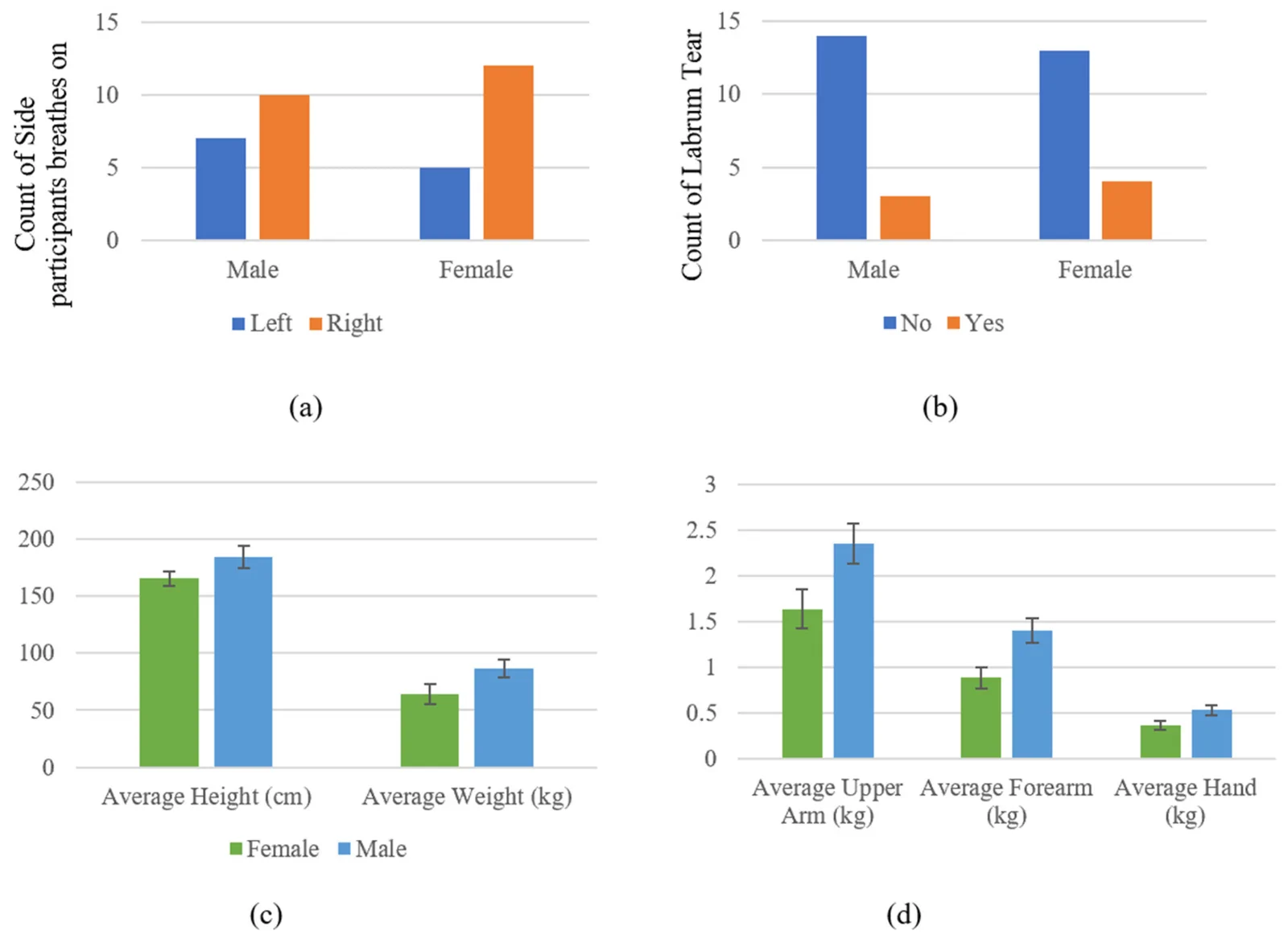
Participant characteristics derived from survey data and presented by sex (female and male). (**a**) Side of breathing (left vs. right); (**b**) prevalence of labral tear; (**c**) average height and weight; and (**d**) estimated average upper-arm, forearm, and hand measurements. For panels (**c**,**d**), data are presented as mean ± standard deviation (SD), with error bars representing one standard deviation from the mean.

**Figure 3:**
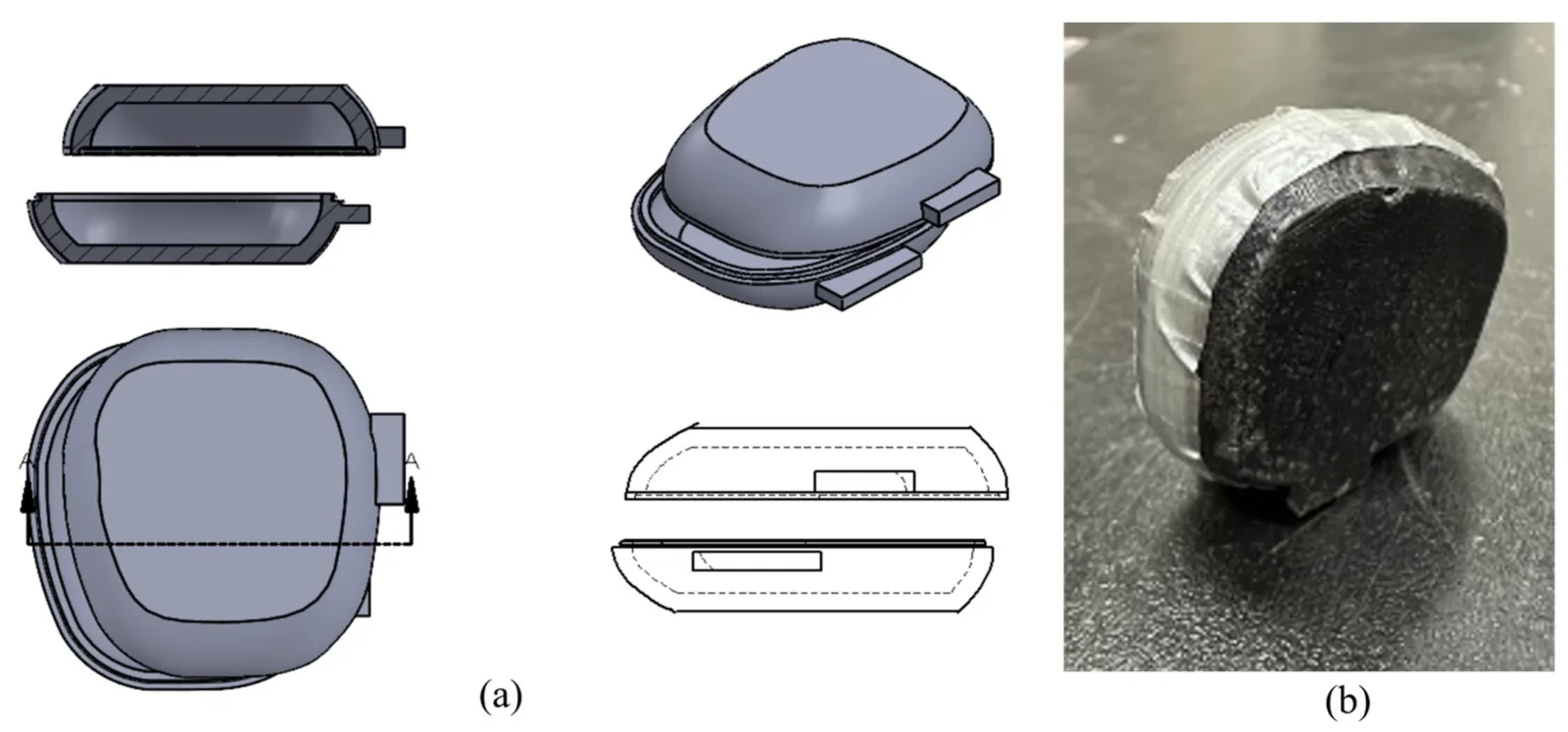
(**a**) SolidWorks CAD design of the waterproof sensor housing. (**b**) Waterproof sensor housing prototype.

**Figure 4:**
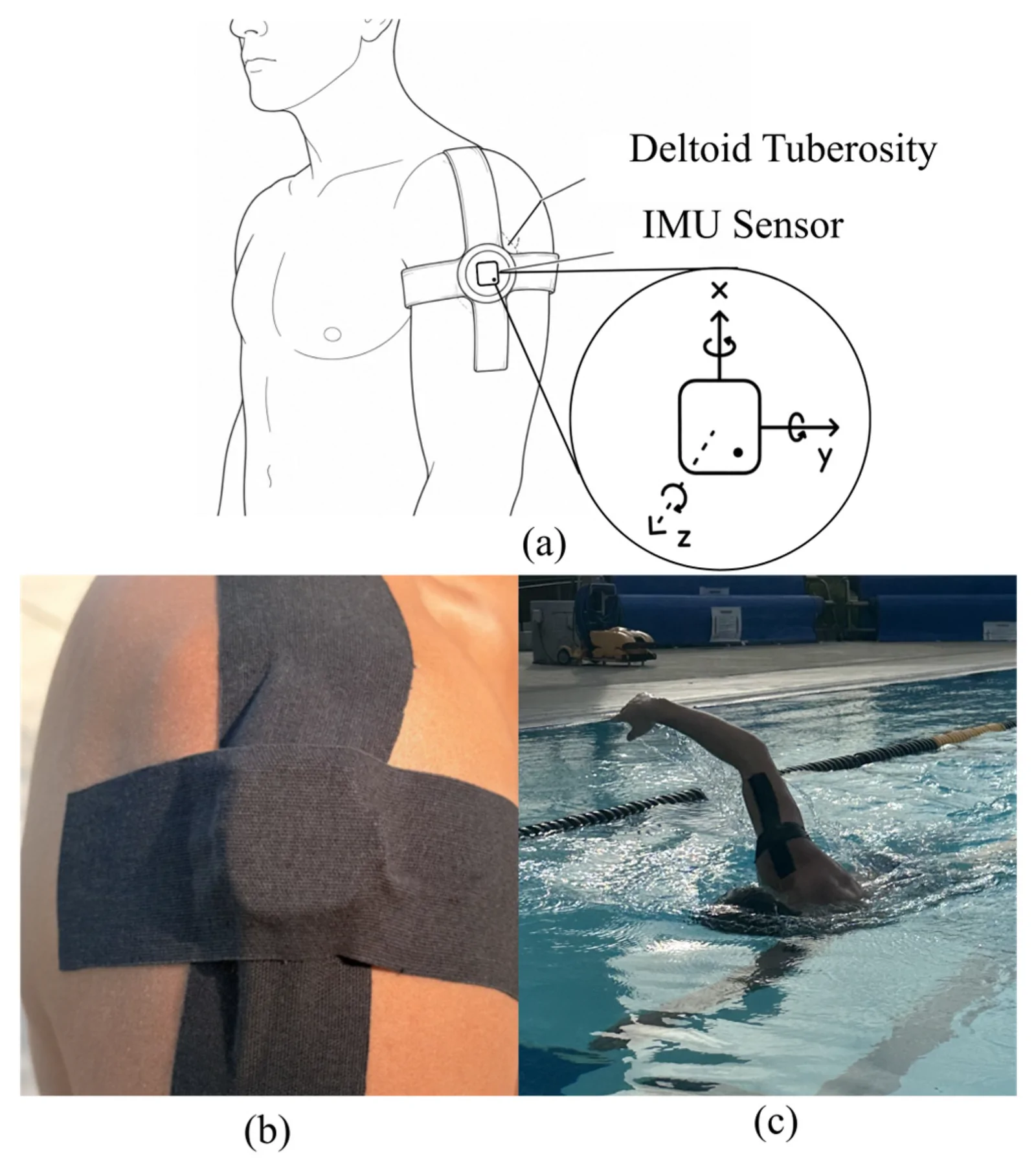
(**a**) Axes configurations for accelerometer and gyroscope components (picture of placement of sensor is generated by ChatGPT). Sensor placement was mirrored on the contralateral limb. (**b**) IMU sensor placement at the deltoid tuberosity of the left shoulder. (**c**) Participant performing data collection protocol with bilateral sensors attached.

**Figure 5:**
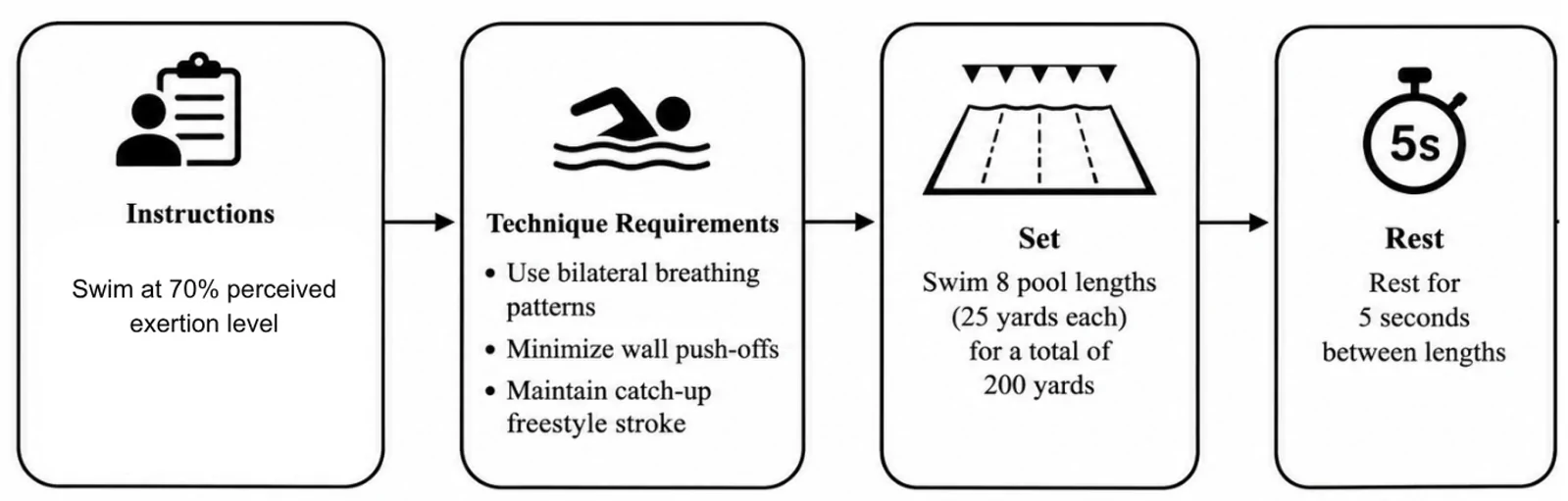
Block diagram of experiment setup for data collection (The block diagram was generated by ChatGPT-5 based on experiment set up).

**Figure 6:**
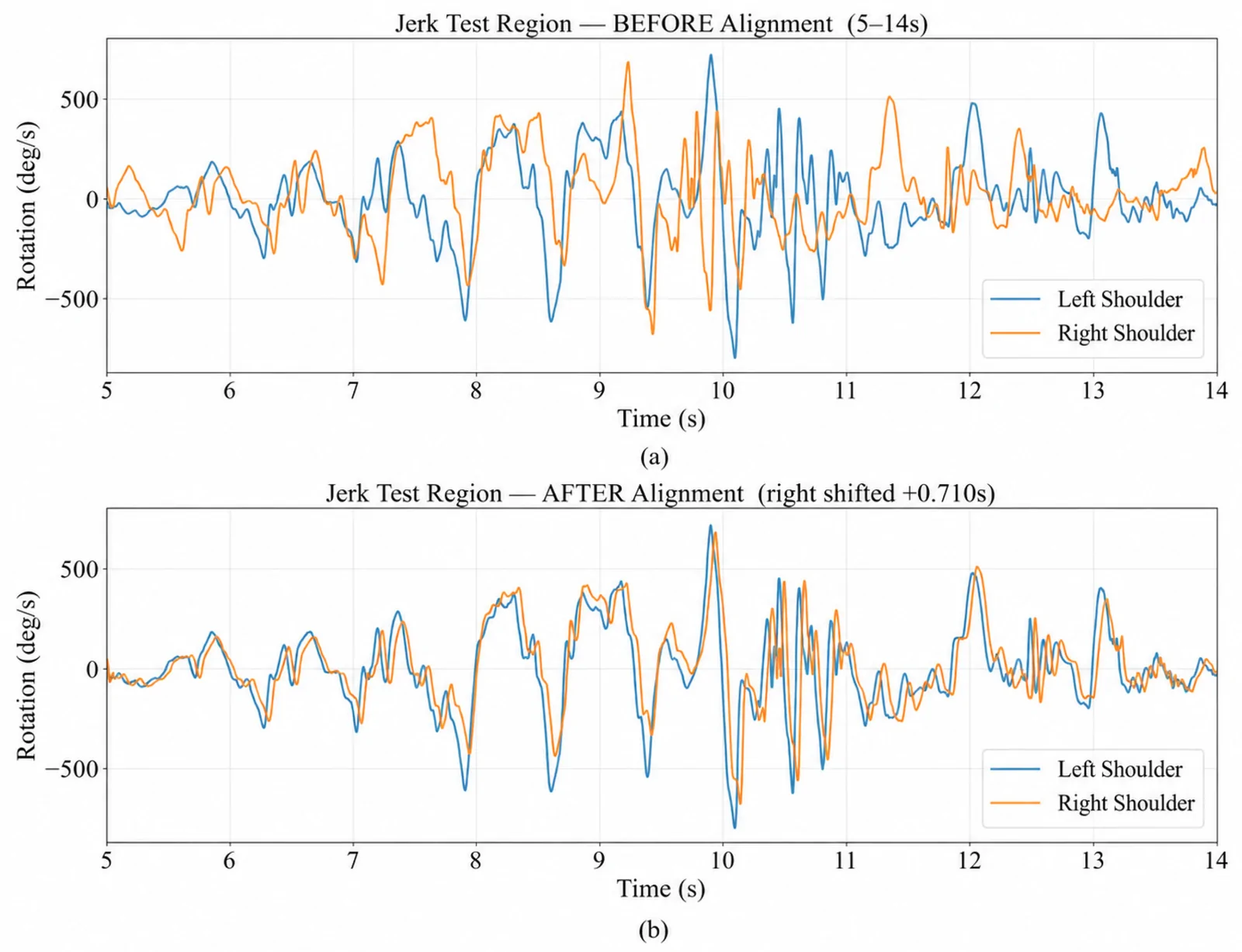
Synchronization of left and right shoulder IMU sensor signals using cross-correlation at the jerk test region. (**a**) Raw signal (**b**) Synchronized.

**Figure 7:**
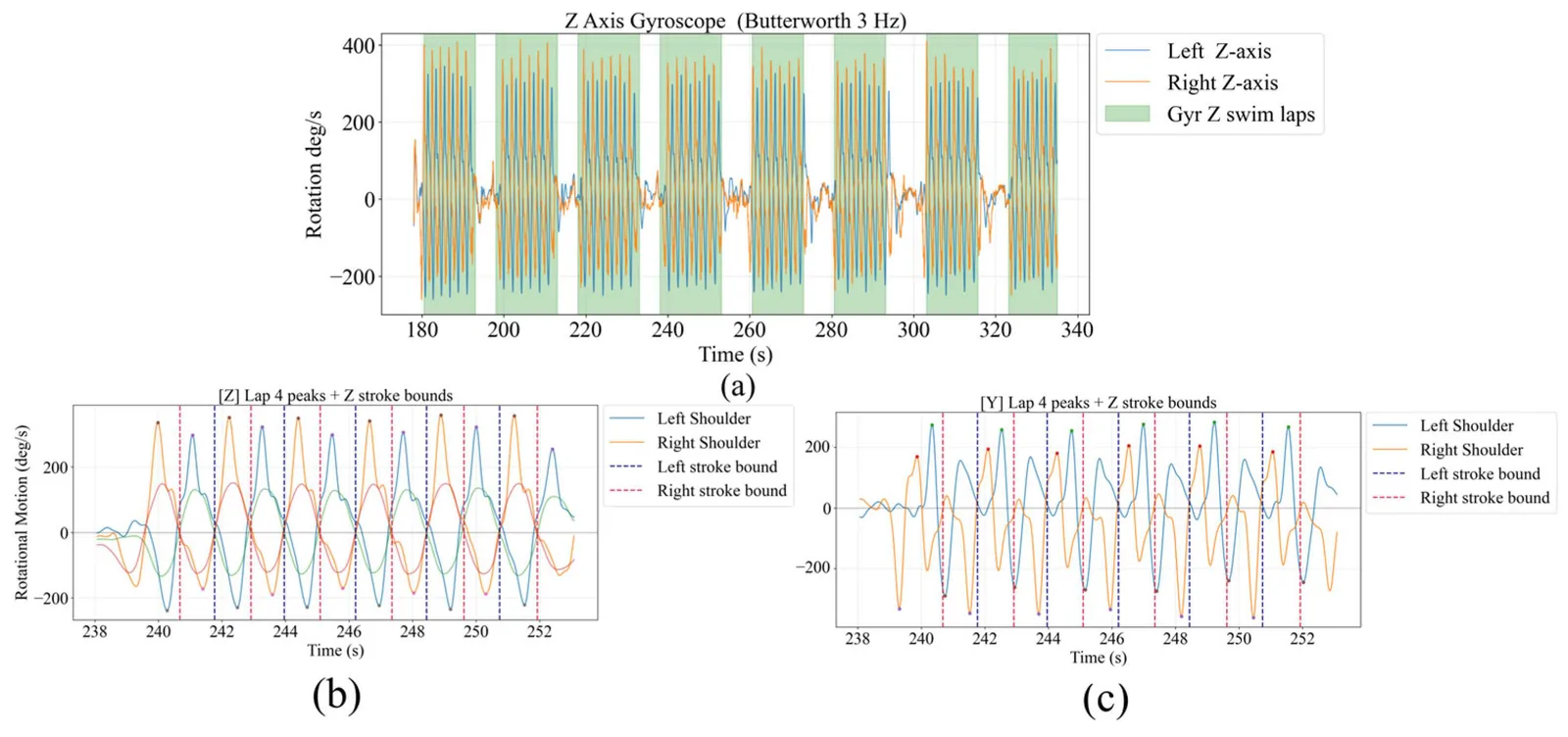
Gyroscope Z-axis rotational motion from bilateral shoulder IMU sensors during swimming to show stroke segmentation pipeline. (**a**) Individual laps (highlighted in green) are detected via individual thresholds, separating swim segments from segments in which the subject is resting between laps. (**b**) The Z-axis gyroscope signal is demeaned via a 201-sample moving average, and falling zero-crossings are marked as stroke boundaries for the left and right signals independently. (**c**) Y-axis gyroscope signal. Stroke boundaries derived from the gyroscope z-axis signal are transferred to corresponding sensors and axes.

**Figure 8:**
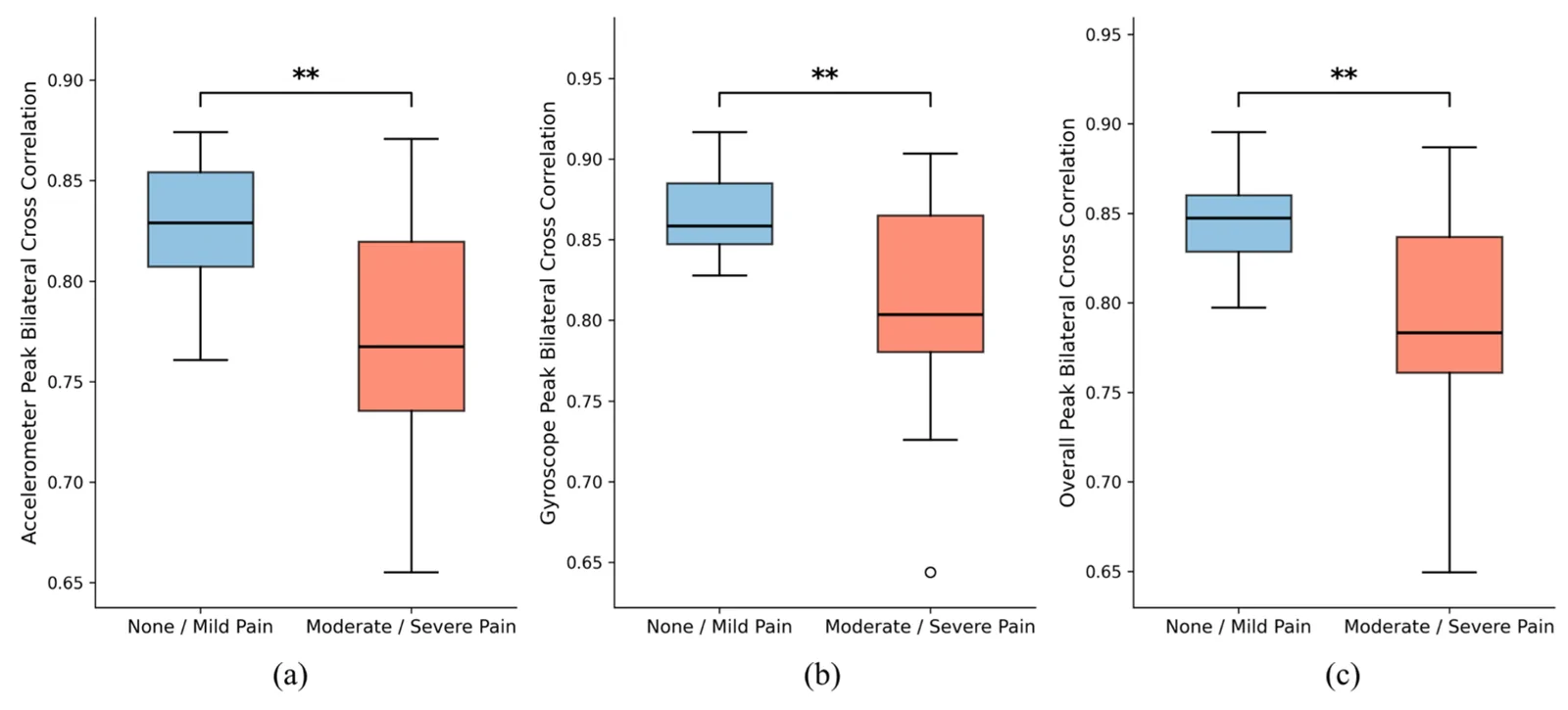
(**a**) Accelerometer Peak Bilateral Cross Correlation (Acc_PeakCorr), (**b**) Gyroscope Peak Bilateral Cross Correlation (Gyr_PeakCorr), (**c**) Overall Peak Bilateral Cross Left-Right Peak Correlation (Ovr_PeakCorr) in moderate/severe pain and none/mild pain groups. Significance bars: Brunner-Munzel two-sided test. ** *p* < 0.01.

**Figure 9:**
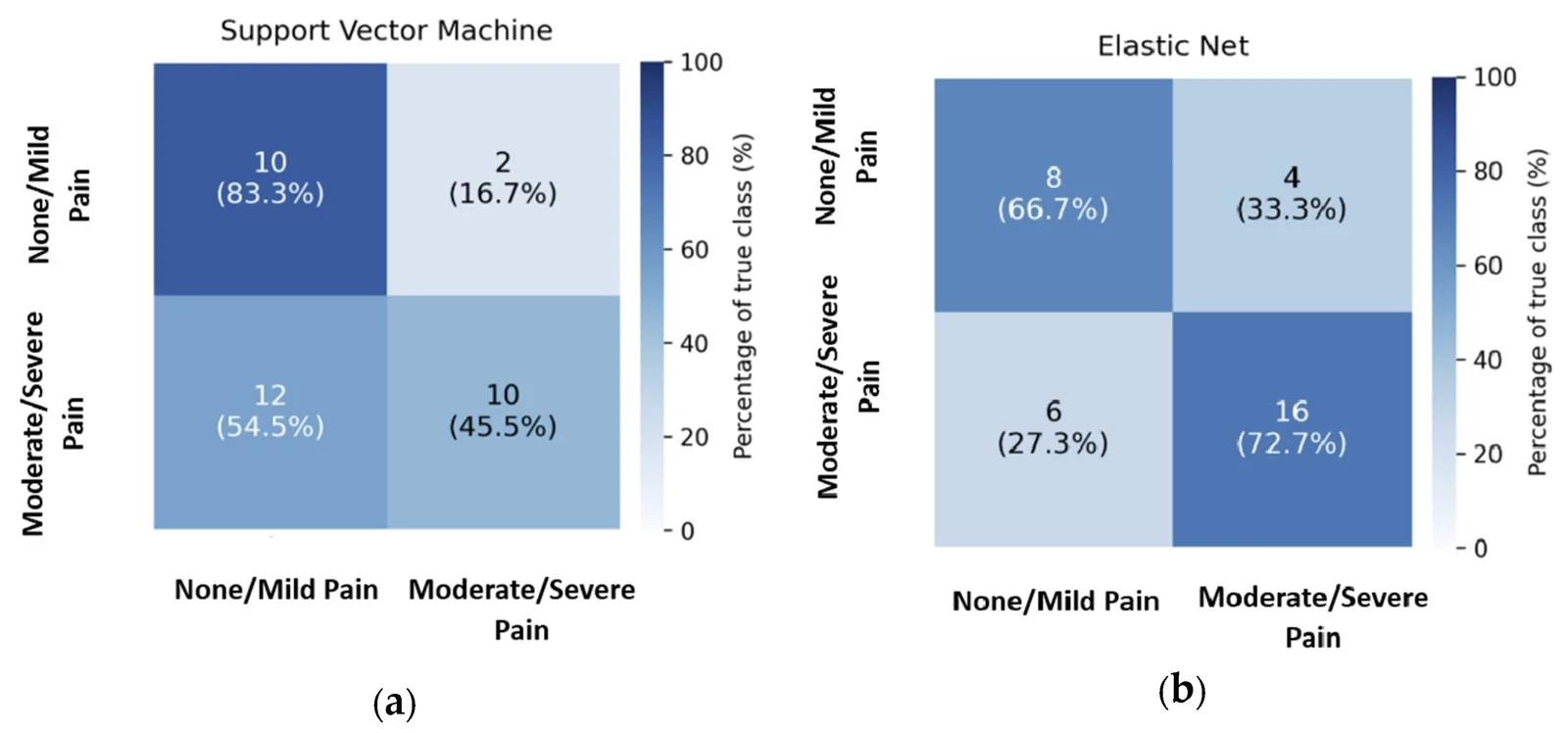
Confusion Matrices for the (**a**) elastic net regression and (**b**) support vector machine models.

**Table 1:** Extracted Features from IMU for Classification of Pain History and Corresponding Abbreviations.

Feature Code Abbreviation	Expanded Definition
Acc_X_PeakCorr	Accelerometer, X-axis: left/right shoulder peak cross-correlation
Acc_Y_PeakCorr	Accelerometer, Y-axis: left/right shoulder peak cross-correlation
Acc_Z_PeakCorr	Accelerometer, Z-axis: left/right shoulder peak cross-correlation
Gyr_X_PeakCorr	Gyroscope, X-axis: left/right shoulder peak cross-correlation
Gyr_Y_PeakCorr	Gyroscope, Y-axis: left/right shoulder peak cross-correlation
Gyr_Z_PeakCorr	Gyroscope, Z-axis: left/right shoulder peak cross-correlation
Acc_PeakCorr	Accelerometer aggregate: peak cross-correlation averaged across all three accelerometer axes (X, Y, Z)
Gyr_PeakCorr	Gyroscope aggregate: peak cross-correlation averaged across all three gyroscope axes (X, Y, Z)
Ovr_PeakCorr	Overall aggregate: peak cross-correlation averaged across both sensors (accelerometer + gyroscope) and all axes (X, Y, Z)

**Table 2:** Statistical Significance of peak bilateral shoulder cross-correlation (PeakCorr) features.

Feature	None/Mild Median (*n* = 12)	Moderate/Severe Pain Median (*n* = 22)	BM Statistic	*p*-Value
Gyr_PeakCorr	0.86	0.80	3.10	0.004
Acc_PeakCorr	0.83	0.77	3.46	0.003
Ovr_PeakCorr	0.85	0.78	3.72	0.002

**Table 3:** Machine learning model classification accuracy metrics.

Model	Accuracy	Balanced Accuracy	ROC AUC
Elastic Net	70.6%	69.7% [52.7–85.2%]	0.731 [0.557–0.890]
SVM	58.8%	64.4% [49.2–79.5%]	0.504 [0.322–0.705]

## Data Availability

The data presented in this study are available on request from the corresponding author.

## Supplementary Materials

The following supporting information can be downloaded at: https://www.mdpi.com/article/10.3390/s26206565/s1, Questionnaire, Anonymous survey data, manually annotated parameters.

## Abbreviations

The following abbreviations are used in this manuscript:

Acc_PeakCorr	Accelerometer aggregate: peak cross-correlation averaged across all three accelerometer axes (X, Y, Z)
Acc_X_PeakCorr	Accelerometer, X-axis: left/right shoulder peak cross-correlation
Acc_Z_PeakCorr	Accelerometer, Z-axis: left/right shoulder peak cross-correlation
AUC	Area Under the Curve
CI	Confidence of Interval
Elastic Net	Elastic Net-regularized logistic regression
Gyr_PeakCorr	Gyroscope aggregate: peak cross-correlation averaged across all three gyroscope axes (X, Y, Z)
Gyr_X_PeakCorr	Gyroscope, X-axis: left/right shoulder peak cross-correlation
Gyr_Y_PeakCorr	Gyroscope, Y-axis: left/right shoulder peak cross-correlation
Gyr_Z_PeakCorr	Gyroscope, Z-axis: left/right shoulder peak cross-correlation
IMU	Inertial Measurement Unit
NCAA	National Collegiate Athletic Association
Ovr_PeakCorr	Overall aggregate: peak cross-correlation averaged across both sensors (accelerometer + gyroscope) and all axes (X,Y,Z)
PeakCorr	Peak Correlation
ROC	Receiver Operating Characteristic
sEMG	Surface electromyography
SVM	Support Vector Machine
