# Supplementary material for: IMU-Based Bilateral Kinematic Waveform Analysis Associated with Shoulder Pain History in Competitive Swimmers
Source: Sensors (Basel). 2026 Oct 16;26(20):6565. doi: 10.3390/s26206565 (PMC13709263; doi:10.3390/s26206565)
Supplement: Supplementary file 1 [file sensors-26-06565-s001.zip › Subject Questionnare.pdf]

# Aquaflow AI Subject Questionnare

**Aug 21, 2026**

1

Name

2

Gender

- ◯ Woman
- ◯ Man
- ◯ Non-binary
- ◯ Prefer not to say

3

Height, in cm

4

Weight in kg

5

Swim Experience

- ◯ 0-2 Years
- ◯ 3-5 Years
- ◯ 5+ years

6

What sport do you play primarily (1= swim) (2=waterpolo)

- ◯ 1
- ◯ 2

7

Do you have history of any shoulder injury

- ◯ Yes
- ◯ No

8

If you breathe predominantly to one side, what side do you breathe to?

- Left
- Right

9

If you have experienced shoulder pain in your swimming career, what side was it on?

- Left
- Right
- Both

10

On a scale from 0-3, how would you describe your shoulder pain?

| Pain Rating | |
|---|---|
| 0 | none |
| 1 | mild (Aware but doesn't change stroke) |
| 2 | Moderate (Might alter stroke/Performance) |
| 3 | Severe (Definetley alters stroke or limits training) |

- 0
- 1
- 2
- 3

11

Have you ever had a confirmed labrum tear in your shoulder?

- ◯ Yes
- ◯ No

This content is neither created nor endorsed by Microsoft. The data you submit will be sent to the form owner.

Microsoft Forms
